# Factors Associated with Improving Appropriate Medical Sharps Disposal Practice Among Diabetic Patients Using Insulin Therapy

**DOI:** 10.12688/f1000research.141978.3

**Published:** 2023-12-08

**Authors:** Ryan Herardi, Hafidz Naeriansyah Djajawiguna, Sri Wahyuningsih, Ida Ayu Kshanti, Shahnaz Medina, Lingga Etantyo Praditya

**Affiliations:** 1 Department of Internal Medicine, Universitas Pembangunan Nasional Veteran Jakarta, Jakarta, Special Capital Region of Jakarta, Indonesia; 2 Undergraduate Program, Universitas Pembangunan Nasional Veteran Jakarta, Jakarta, Special Capital Region of Jakarta, Indonesia; 3 Department of Public Health, Universitas Pembangunan Nasional Veteran Jakarta, Jakarta, Special Capital Region of Jakarta, Indonesia; 4 Department of Internal Medicine, Fatmawati General Hospital, South Jakarta, Special Capital Region of Jakarta, Indonesia; 5 Research Staff Faculty of Medicine, Universitas Pembangunan Nasional Veteran Jakarta, Jakarta, Special Capital Region of Jakarta, Indonesia

**Keywords:** Keywords: Diabetes, Insulin, Medical sharp product, Disposal Practice, Formal Training

## Abstract

**Background:**

Diabetic patients are always in contact with medical sharps, such as pen needles, lancets, and syringes. Sometimes, patients improperly dispose of these items and cause needle stick injuries. This study aimed to identify factors that improve appropriate manner in which individuals with diabetes who require insulin therapy dispose of medical sharps.

**Methods:**

In December 2019, a cross-sectional investigation was undertaken amongst insulin therapy-dependent diabetic patients visiting Jakarta’s Fatmawati General Hospital. A questionnaire was formulated to appraise medical sharps’ disposal procedure. The data gathered the questionnaire, including age, gender, educational level, employment status, length of time as a diabetic, duration of insulin treatment, and receipt of formal medical training, were also recorded.

**Results:**

Of 103 diabetic patients, 77.3% were over 50 years old, 58.3% were female, 68% were a low level of education, 74.8% were not working, 84.5% were diagnosed with diabetes for more than 5 years, 53.4% were using insulin therapy for more than 5 years, and only 65% had got formal training on medical sharp products disposal. Nearby 83.5% still recap the pen needle insulin with the inner needle cap after injecting insulin, and 92.2% still threw medical sharps on the street when traveling outside. Approximately 81.6% of respondents stored their unused needles and lancets in a secured manner that was inaccessible to children. The practice score for proper medical sharps disposal increased from 4.5 to 6.0 as a result of formal training provided by healthcare professionals, as determined by the Mann Whitney Test (p=0.001).

**Conclusions:**

Formal training by healthcare workers was the only factor that improved medical sharps disposal practice among diabetic patients using insulin therapy.

## Introduction

The medical condition known as “Diabetes Mellitus (DM)” is categorized as a metabolic disease that presents with hyperglycemia. The manifestation of this chronic condition results from malfunctions in either insulin secretion, insulin action, or a combination of both. ^1^ In the years between 2007 and 2010, a significant proportion of individuals - 88.2% of those aged 20 years and above - diagnosed with this condition in the United States sought to manage their symptoms by administering insulin and/or oral medications. Further analysis revealed that within this group, 58.4% relied solely on oral medications, 15.3% only utilized insulin, while 14.5% adopted a combination therapy involving both insulin and oral medications. ^2^

Patients with diabetes who utilize insulin therapy are constantly in contact with medical sharps, including lancets, pen needles, and syringes. Regrettably, patient disposal of these sharps is not always adequate. The presence of medical sharps poses a considerable risk to workers in the waste management sector, as well as janitors, refuse collectors, and members of adjacent communities who are exposed to these materials. This issue is especially concerning, as the individuals are at risk of needle stick injury and contracting infectious diseases such as hepatitis B, hepatitis C, and Human Immunodeficiency Virus (HIV). ^3^^–^^5^

A study in Gondar Town, Ethiopia displayed a correlation between proper disposal of insulin injection medical waste and urban residency, high educational attainment, a diagnosis of type 1 diabetes, insulin use duration of less than 5 years, and physician visits within the last six months. ^4^ Conversely, another study performed in New Delhi, India did not identify any connection between knowledge, socio-demographic factors, and attitude regarding household sharp waste disposal and adherence to proper disposal of sharp waste. ^5^ A study conducted in a tertiary hospital in New York, United States, demonstrated that individuals with diabetes for more than 30 years and who received formal training in sharps disposal exhibited a greater likelihood of exercising correct practices. ^6^ Similarly, a report from North-East Peninsula Malaysia describes a correlation between previous guidance on sharp disposal from healthcare providers, patients’ knowledge of safe disposal, and the duration of diabetes being less than five years with proper sharp waste disposal. ^7^ In Sri Lanka, a research study highlighted that level of education, duration of insulin usage, and prior education on safe disposal were all independently associated with proper disposal of sharps. ^8^

## Methods

A cross-sectional study of convenience sample was conducted to identify factors that improve appropriate medical sharps disposal practices among diabetic patients. The “sharp” related Diabetes Mellitus includes needles, lancets, and syringes. The samples used in this study were 103 patients with diabetes mellitus who were using insulin therapy and met the inclusion and exclusion criteria. Inclusion and exclusion criteria are listed below:

a. Inclusion criteria
  1) Type 2 diabetes mellitus patients who are currently using insulin therapy.
  2) Type 2 diabetes mellitus patients in outpatient treatment.
b. Exclusion criteria
  1) Patients who are not willing to participate in the study.
  2) Patients who did not complete the questionnaire completely.

Data was collected from a questionnaire asking type 2 DM patients who visited Fatmawati General Hospital, Jakarta, Indonesia, on December 2019.

In order to evaluate the disposal of medical sharps, we constructed a questionnaire following Singh, ^5^ and modified it based on recent literature on proper medical sharps disposal recommended by the Centers for Disease Control and Prevention (CDC), American Diabetes Association (ADA) and National Health Service (NHS) United Kingdom (UK). ^9^^–^^12^ Twelve questions were translated into Bahasa Indonesia and conducted with 30 subjects to validate and assess the reliability of the questions. For each question, dichotomous scoring was applied, where one mark was awarded for a correct response and zero for an incorrect response. A score of zero indicated the poorest practice, while a score of 12 demonstrated the best practice.

In the initial stage, we sorted patients currently using insulin needles. Patient data was obtained from existing hospital records, allowing us to identify those using insulin needles and those who were not. Subsequently, we contacted these patients and provided instructions directly, covering everything from obtaining informed consent to the procedure for filling out the questionnaire. We assisted respondents directly in completing the questionnaire to minimize errors in the process.

The study collected data on various factors associated with the disposal of medical sharps, including age, gender, level of education, employment status, duration of diabetes, duration of insulin use, and formal training from healthcare workers. Age was categorized using a threshold of 50 years, while gender was classified as male or female. Level of education was grouped into low (patients with no schooling, elementary, junior high, and high school education) and high (patients with a diploma, undergraduate, postgraduate, or doctorate) categories. Employment status was classified as actively employed or not working (including retired individuals). The duration of diabetes and insulin use were used as cut-off points of five years each. Additionally, patients were asked about any training they received from doctors, nurses, or other healthcare workers prior to disposing of sharp medical products.

Before being analyzed by IBM ^®^ SPSS ^®^ (Statistical Package for the Social Sciences) software version 26.0 for Mac, the last data collection tool was thoroughly checked for completeness. Univariate analysis employed frequencies and percentages to represent different variables, while bivariate analysis utilized the Mann-Whitney test to compare groups. The statistical tests were carried out at a significance level of 0.05.

Prior to conducting this study, each participant was given the opportunity to provide written informed consent. Additionally, this study received approval from “The Health Research Ethics Committee of Universitas Pembangunan Nasional Veteran Jakarta” (UPNVJ), Jakarta, Indonesia (Registration Number: B/2228/XII/2019/KEPK). We used the STROBE cross sectional checklist when writing our report. ^13^

## Results

The result in Table 1 pertains to diabetic patients currently undergoing insulin therapy. Most of these patients were over 50 years old (77.3%), female (58.3%), and had a low level of education (68.0%). Additionally, most of the patients were not working (74.8%) and were diagnosed with diabetes for more than five years (84.5%). Among those who had undergone insulin therapy for more than 5 years, 55 individuals were identified. Regarding formal training received from healthcare workers, most patients (65.0%) reported having received any training regarding proper medical sharp disposal.

**Table 1.** Diabetic patients’ characteristic.

Variables	n	%
**Age**		
≥ 50 years old	80	77.7
< 50 years old	23	22.3
**Gender**		
Male	43	41.7
Female	60	58.3
**Level of education**		
High	33	32.0
Low	70	69.0
**Working status**		
Actively employed	26	25.2
Not working	77	74.8
**Duration of diabetes mellitus**		
Less than 5 years	16	15.5
5 years or more	37	84.5
**Duration of insulin therapy**		
Less than 5 years	48	46.6
5 years or more	55	53.4
**Formal training from healthcare workers**		
Yes	67	65.0
No	36	35.0
**Total**	103	100.0

Table 2 shows the percentage of right answers from each question for evaluating medical sharps disposal. The majority of diabetic patients still recap the pen needle insulin with an inner needle cap after injecting insulin (83.5%), even though it was no longer recommended due to the potential for needle stick injury. In addition, although many patients collected medical sharps into a special container, they still threw sharp medical products on the street when traveling outside (92.2%). Most patients kept their unused needles and lancets in a safe place and were unreachable to children (81.6%).

**Table 2.** Percentage of patients' practices regarding medical sharps disposal.

No	Questions	Yes (%)	No (%)
1	I recap the pen needle insulin with small / inner needle cap after injecting insulin ^*^	83.5	16.5
2	I throw away insulin needle and lancets into the household plastic bags ^*^	51.5	48.5
3	Sometimes I collect sharp waste in plastic containers or tin cans.	55.3	44.7
4	I sometimes re-use pen needle insulin more than 1 day, if the condition seems right to use again. ^*^	73.8	26.2
5	If I go out, I bring my used needles and lancets back home.	60.2	39.8
6	I throw sharps on street if I am travelling outside or in a party. ^*^	92.2	7.8
7	I bend the needle and sharp after use so that it cannot be re-used by anyone else. ^*^	46.6	53.4
8	I keep my unused needles and lancets at a place not reachable to children and others	81.6	18.4
9	I collect all sharps and dispose at one particular day.	52.4	47.6
10	I have informed my garbage picker of sharps in my garbage.	34.0	66.0
11	I have asked the doctor about disposal of pen needles and lancets	48.5	51.5
12	I have asked the pharmacist about disposal of pen needles and lancets.	45.6	54.4

* Negative statement (answering “No” scored 1).

Table 3 describes the results of the association between influencing factors and disposal practice scores. Training from health workers was associated with improving medical sharps disposal patients among diabetic patients (p=0.001; Mann Whitney Test). Other factors were not significantly associated.

**Table 3.** Associations between influencing factors and disposal practice.

Factors	Disposal Practice Score Median (IQR)	p ^*^
**Age**		
≥ 50 years old	5.5 (2.0)	0.20
< 50 years old	5.0 (3.0)	
**Gender**		
Male	6.0 (3.0)	0.49
Female	5.0 (3.0)	
**Level of education**		
High	5.0 (3.0)	0.56
Low	5.0 (3.0)	
**Employment status**		
Actively employed	5.0 (3.0)	0.66
Not Working	5.0 (3.0)	
**Duration of diabetes**		
Less than 5 years	6.5 (3.0)	0.66
5 years or more	5.0 (3.0)	
**Duration of insulin use**		
Less than 5 years	5.0 (3.0)	0.84
5 years or more	5.0 (3.0)	
**Formal training from health workers**		
Yes	6.0 (3.0)	0.01
No	4.5 (3.0)	

IQR = Interquartile Range.

^*^ Mann Whitney Test.

## Discussion

The diabetic patients who use insulin exhibited significant diversity regarding their age, gender, level of education, employment status, duration of diabetes, duration of insulin use, and formal training from healthcare workers. Only two-thirds of diabetic patients admitted to having training related to disposing of proper medical sharps by healthcare workers. It showed that we still have a lot of opportunities to educate diabetic patients more creatively.

Almost all diabetic patients still recap the pen needle insulin with an inner needle cap after injecting insulin. American Diabetes Association, CDC, and NHS UK no longer recommend it due to the potential for needle stick injury. Similar results were reported by Mecuria, Singh, Atukorala, and Montoya. ^4^^,^^5^^,^^8^^,^^14^ Diabetic patients should be advised to cover the insulin pen only with the pen cap or to throw it away without closing the container when no longer used. ^9^^–^^12^ Only half of the diabetic patients threw medical sharps into separate containers. The rest threw them directly into household plastic bags. Singh reported almost all patients threw them directly into household garbage bags. ^5^ This can endanger the dustman to getting a needle stick injury. When traveling out of the house, most diabetic patients would bring used needles and lancets back home, but if traveling far away, almost all patients threw them out of hand. Singh found that almost all patients did not throw sharps on the street, but they did not bring them back home either, so that finding was unclear. ^5^ Patients’ awareness of bringing used medical sharps back home needs to be increased by reminding them to carry a specific container when they are traveling. Most patients still reuse insulin pen needles for more than one day. American Diabetes Association recommended changing the needle after each injection or at least once daily. ^9^ This may occur because of the limited free pen needles covered by National Insurance. They need extra money to buy pen needles and lancets. Singh reported a similar study in India. ^5^

Most of the patients no longer bent the needle after using it. This is a positive behavior recommended by American Diabetes Association, CDC, and NHS UK. ^9^^–^^12^ In contrast, Singh and Mecuria found that more than half of patients still bent the needle and lancet. ^4^^,^^5^ Concern about keeping sharp medical products out of reach of children at home was quite good, similar to that reported in another study by Singh. ^5^ Half of the diabetic patients had regularly disposed of containers containing medical sharps on one particular day, but the majority of patients had never informed the dustman about the existence of sharp medical products in their waste. Different from our study, Singh found almost all patients did not dispose of them on one particular day and also did not inform the dustman. This may be related to the absence of national regulation regarding medical waste disposal in household waste. ^5^ Only half of the diabetic patients actively asked doctors or pharmacy staff about the appropriate way to dispose of pen needles and lancets. Singh and Mecuria found less than a quarter of patients had asked about the appropriate way to dispose of medical sharps. ^4^^,^^5^ This makes us need to be more active in providing information about how to properly dispose of medical sharps, especially diabetic-related sharp waste.

This study showed that the only factor associated with improving medical sharps disposal practice was formal training from healthcare workers (p=0.01; Mann-Whitney Test), while other factors were not. This is similar to the study conducted by Singh, ^5^ education by Health Care Providers was associated with good practice on disposal of sharp waste, while knowledge and attitude levels were not. Singh also found an association between good practice on the disposal waste with the education given by pharmacists and friends, not only from health care providers. Huang found the same results that “formal training on proper sharps disposal was more likely to dispose of sharps correctly, and other factors were not associated.” ^6^ Hasan and Atukorala found similar results that “previous advice on sharp disposal from health care providers was significant contributing factor for sharp waste disposal.” ^7^^,^^8^ They also found that “the duration of diabetes (less than five years) was a significant contributing factor due to chronic disease burnout.” ^7^^,^^8^ Montoya found similar results that “patients who disposed of needles in an unsafe manner had DM for a longer duration than those who used safe disposal patterns.” ^14^ Similar to our findings, Instead of using numerical values, we categorized the length of time someone has had diabetes into less than five years or more than five years. Practicing diabetes management for a shorter time frame of less than five years was found to be more advantageous than practicing for a longer duration of more than five years, although this difference was not statistically significant and may be due to the smaller sample size. Huang, Hasan, Atukorala, and Montoya discovered that “formal training on proper sharps disposal was strongly associated with correct disposal, while other factors showed no significant association.” ^6^^–^^8^ Moreover, they found that previous advice on sharp disposal from healthcare providers was a significant contributing factor to proper waste disposal. ^6^^–^^8^ Hasan and Atukorala also identified “the duration of diabetes (less than five years) as a significant contributing factor due to chronic disease burnout.” ^7^^,^^8^ Montoya observed similar findings, indicating that “patients who disposed of needles unsafely had been diagnosed with DM for a longer duration than those who used proper disposal practices.” ^14^ Our study revealed similar results, suggesting that patients with diabetes for less than five years tend to exhibit better disposal practices than those with a longer duration of diabetes, but the difference was not statistically significant. This might be attributed to the smaller sample size and the categorization of the duration of diabetes as less than or more than five years rather than using a numerical value.

According to Mecuria’s study, the factors that significantly affect the proper disposal of insulin injection devices were living in urban areas, educational status, type 1 DM, duration of insulin use, and frequency of physician visits. However, they did not find a direct association between healthcare worker training and good disposal practices. Our study agreed with Mecuria, but they were different considering that we did not divide our patients’ residence (they live in a homogenous area), type of diabetes (almost all of them were type 2 DM patients), and frequency of physician visit (chronic patients were required to visit once a month due to National Insurance regulation). Mecuria also divided education level and duration of insulin use in more detail. ^4^

The role of repeated formal training from healthcare workers regarding appropriate medical sharps disposal properly is very crucial. A study conducted by Hasan showed “there was a significant increase in the mean knowledge score” after providing health education interventions, as observed at one-month and three-month follow-up assessments. ^15^ The government should formulate a specific regulation regarding the management of medical waste in households. Before the existence of an official regulation, health facilities could persuade patients to collect their medical waste and bring it back to their health facilities that have incinerators to destroy them properly.

We recommend conducting training on the proper disposal of insulin needles. The training can be carried out regularly on an individual basis, with a frequency of once a month, aligning with patients’ scheduled check-ups with the internal medicine specialist. Additionally, group training can be organized through health seminars for users at Fatmawati Hospital. We strongly advocate for training on the correct and safe disposal of insulin needles to reduce the risk of disease transmission due to needlestick incidents.

## Limitations

While this investigation provides valuable insights into the Factors Associated with Improving Appropriate Medical Sharps Disposal Practice Among Diabetic Patients Using Insulin Therapy, it is imperative to acknowledge the inherent limitations that shape the scope and interpretation of our findings. These limitations, discussed below, underscore the need for cautious interpretation and serve as a foundation for future research endeavors in this domain.

**Cross-Sectional Design:** This study employed a cross-sectional design, capturing a snapshot of data at a single point in time. Consequently, the inherent limitation of not being able to establish causal relationships between variables exists. Future research employing longitudinal or interventional designs is essential to elucidate causal connections and observe changes over time.

**Training Standardization Variation:** Healthcare workers provide complete formal training including how to use insulin, how to check blood sugar independently, and how to dispose of medical sharp devices, but there is no detailed standard yet of what should be conveyed by health workers so that the delivery of training and patient capture can vary.

**Inability to Explore Evolution of Skills:** The study did not investigate the evolution of skills over time, capturing information at a single time point. Examining the long-term retention of skills acquired during training could provide a more comprehensive understanding of the effectiveness of healthcare workers' education programs.

## Conclusion

Almost all diabetic patients still recap the pen needle insulin with a small cap after injecting insulin and throw sharp medical products on the street when traveling outside. Most diabetic patients who used insulin therapy stored their unused needles and lancets in a secure location that was inaccessible to children. Notably, the only factor that was found to have a positive impact on medical sharps disposal practices among these patients was formal training provided by healthcare workers.

## Data Availability

Figshare: Questionnaire based on collection of socioeconomic, knowledge, attitude and presence of influencing factors, DOI: https://doi.org/10.6084/m9.figshare.22735724. This project contains the following underlying data:
• Questionnaire based on collection of socio-economic, knowledge, attitude and presence of influencing factors modified based on recent literature on proper medical sharps disposal recommended by the Centers for Disease Control and Prevention (CDC), American Diabetes Association (ADA) and National Health Service (NHS) United Kingdom (UK). Data are available under the terms of the Creative Commons Attribution 4.0 International license (CC-BY 4.0).
