# Supplementary material for: Factors Associated with Improving Appropriate Medical Sharps Disposal Practice Among Diabetic Patients Using Insulin Therapy
Source: F1000Res. 2023 Dec 8;12:1290. Originally published 2023 Oct 9. [Version 3] doi: 10.12688/f1000research.141978.3 (PMC10719556; doi:10.12688/f1000research.141978.3)
Supplement: Peer review history

# Reviewer report: Reviewer response for version 3

**DOI:** 10.5256/f1000research.159706.r228705

**Published:** 2023-12-13

**Authors:** Ahmad Fariz Malvi Zamzam Zein

**Affiliations:** 1 Department of Internal Medicine, Waled General Hospital, Cirebon, West Java, Indonesia; 2 Department of Internal Medicine, Faculty of Medicine, University of Swadaya Gunung Jati, Cirebon, West Java, Indonesia

I have no further comments.

Is the work clearly and accurately presented and does it cite the current literature?

Yes

If applicable, is the statistical analysis and its interpretation appropriate?

Yes

Are all the source data underlying the results available to ensure full reproducibility?

Yes

Is the study design appropriate and is the work technically sound?

Yes

Are the conclusions drawn adequately supported by the results?

Yes

Are sufficient details of methods and analysis provided to allow replication by others?

Yes

Reviewer Expertise:

Internal Medicine, Epidemiology

I confirm that I have read this submission and believe that I have an appropriate level of expertise to confirm that it is of an acceptable scientific standard.

# Reviewer report: Reviewer response for version 2

**DOI:** 10.5256/f1000research.158913.r224727

**Published:** 2023-11-27

**Authors:** Ahmad Fariz Malvi Zamzam Zein

**Affiliations:** 1 Department of Internal Medicine, Waled General Hospital, Cirebon, West Java, Indonesia; 2 Department of Internal Medicine, Faculty of Medicine, University of Swadaya Gunung Jati, Cirebon, West Java, Indonesia

Abstract

 In Methods section:

1. It is written as 'A questionnaire was formulated to appraise medical sharps' disposal methodology'. It is preferable to replace 'methodology' with other relevant word, such as method, procedure, or practice.
2. it is written as 'The data gathered from the said patients, ...'. What is the meaning of 'the said patients'? The study used assisted questionnaire for data collecting.

Discussion

1. In paragraph 2, it is wiritten as 'ADA, CDC, and NHS UK no longer recommend it ...' It is preferable to replace ADA with non-abbreviated relevant word, regarding the writing in the beginning of sentence.
2. In paragraph 2, it is written as 'Similar studies were reported by ....' and 'Singh reported a similar study in India' It is preferable to replace 'studies' or 'study' with other relevant word, such as result or finding.
3. Authors did not mention the limitation of the study.

Writing

1. It is highly recommended to use the same terms or nomenclatures consistently. Otherwise, it is optional to use abbreviation for making it efficiently. For example, in the manuscript, it is written medical sharp product (in keyword), sharp(s) (in introduction section) and sharp medical products (in methods section).

Overall, this study emerges the urgency of the problem properly. To my best knowledge, it is the first study in Indonesia investigating the practice of medical sharps disposal. The method used in the study was adequate and the discussion was contented.

Is the work clearly and accurately presented and does it cite the current literature?

Yes

If applicable, is the statistical analysis and its interpretation appropriate?

Yes

Are all the source data underlying the results available to ensure full reproducibility?

Yes

Is the study design appropriate and is the work technically sound?

Yes

Are the conclusions drawn adequately supported by the results?

Yes

Are sufficient details of methods and analysis provided to allow replication by others?

Yes

Reviewer Expertise:

Internal Medicine, Epidemiology

I confirm that I have read this submission and believe that I have an appropriate level of expertise to confirm that it is of an acceptable scientific standard, however I have significant reservations, as outlined above.

# Reviewer report: Reviewer response for version 2

**DOI:** 10.5256/f1000research.158913.r223386

**Published:** 2023-11-16

**Authors:** Velma Herwanto

**Affiliations:** 1 Faculty of Medicine, Tarumanagara University, West Jakarta, Jakarta, Indonesia; 2 Internal Medicine, Siloam Hospitals Kebon Jeruk, West Jakarta, Jakarta, Indonesia

No further comment.

Is the work clearly and accurately presented and does it cite the current literature?

Yes

If applicable, is the statistical analysis and its interpretation appropriate?

Yes

Are all the source data underlying the results available to ensure full reproducibility?

Yes

Is the study design appropriate and is the work technically sound?

Yes

Are the conclusions drawn adequately supported by the results?

Yes

Are sufficient details of methods and analysis provided to allow replication by others?

Yes

Reviewer Expertise:

Internal medicine, sepsis, infectious diseases

I confirm that I have read this submission and believe that I have an appropriate level of expertise to confirm that it is of an acceptable scientific standard.

# Reviewer report: Reviewer response for version 1

**DOI:** 10.5256/f1000research.155469.r213222

**Published:** 2023-10-16

**Authors:** Velma Herwanto

**Affiliations:** 1 Faculty of Medicine, Tarumanagara University, West Jakarta, Jakarta, Indonesia; 2 Internal Medicine, Siloam Hospitals Kebon Jeruk, West Jakarta, Jakarta, Indonesia

Abstract:

-  "53.4% were using insulin therapy" --> what does this refer to? All of the participant used insulin therapy.

Methods:

- This article has not clearly said the inclusion and exclusion criteria of their participant. I suppose the inclusion criterion was diabetes patient on insulin therapy.
- Authors have not explained the procedure to fill out the questionnaire in detail. If, for example, the participant faced difficulty to fill it out (might be due to visual problem or low education), what did he researcher do to make it work?

Discussion:

- "Singh found that almost patients did not throw sharps on the street, but ..." --> I would guess there is an "all" after the word almost.
- Author could elaborate more on how the formal training was done by the health workers. For example, if the training was conducted routinely in Fatmawati Hospital, it might be useful to explain the training a bit more (the frequency, in group or in private, etc).

References:

- Bibliography needs to be fixed, including the punctuation, title sentences. References need to be completely written (eg, reference 9 and 10 need to be corrected).

Is the work clearly and accurately presented and does it cite the current literature?

Yes

If applicable, is the statistical analysis and its interpretation appropriate?

Yes

Are all the source data underlying the results available to ensure full reproducibility?

Yes

Is the study design appropriate and is the work technically sound?

Yes

Are the conclusions drawn adequately supported by the results?

Yes

Are sufficient details of methods and analysis provided to allow replication by others?

Yes

Reviewer Expertise:

Internal medicine, sepsis, infectious diseases

I confirm that I have read this submission and believe that I have an appropriate level of expertise to confirm that it is of an acceptable scientific standard.

# Response

**Published:** 2023-11-13

**Authors:** Ryan Herardi

**Affiliations:** University of Pembangunan Nasional Veteran jakarta, Indonesia

Dear dr. Velma Herwanto,

 Thank you for your constructive feedback on our manuscript titled “Factors Associated with Improving Appropriate Medical Sharps Disposal Practice Among Diabetic Patients Using Insulin Therapy”

 We appreciate the thoroughness of your review, and we have addressed each of your concerns below:

 Comment 1:

 In the revised manuscript, we will clarify this point by specifying the inclusion and exclusion criteria in the Methods section. The inclusion criteria now explicitly state that participants are Type 2 diabetes mellitus patients currently using insulin therapy, and the exclusion criteria outline conditions such as unwillingness to participate or incomplete questionnaire submission.

 Comment 2:

 We appreciate your observation, and to address this concern, we are going to include detailed inclusion and exclusion criteria in the Methods section, specifying the eligibility of Type 2 diabetes mellitus patients currently using insulin therapy as participants.

 Comment 3:

 In response to your comment, we are going to add information in the Discussion section explaining the steps taken during the questionnaire administration. We identified patients using insulin needles from hospital records, provided direct instructions, obtained informed consent, and assisted respondents in completing the questionnaire to minimize errors.

 Comment 4:

 Your observation is correct. We are going to amend the sentence to read, "Singh found that almost all patients did not throw sharps on the street."

 Comment 5:

 To address this suggestion, we are going to include additional details in the Discussion section, recommending regular individual training sessions, monthly frequency aligned with patients' check-ups, and the possibility of group training through health seminars at Fatmawati Hospital.

 Comment 6:

 We have thoroughly reviewed and revised the bibliography, ensuring correct punctuation and complete references, including the correction of references 9 and 10.

 We believe these revisions strengthen the clarity and completeness of our manuscript. We sincerely appreciate your valuable input, which has contributed to enhancing the overall quality of our work.

 Thank you for your time and diligence in reviewing our manuscript.

 Best Regards,

 Author
